# Discovery and characterisation of circular bacteriocin plantacyclin B21AG from *Lactiplantibacillus plantarum* B21

**DOI:** 10.1016/j.heliyon.2020.e04715

**Published:** 2020-08-21

**Authors:** Aida Golneshin, Mian-Chee Gor, Nicholas Williamson, Ben Vezina, Thi Thu Hao Van, Bee K. May, Andrew T. Smith

**Affiliations:** a School of Sciences, RMIT University, Bundoora, Victoria, Australia; b Edlyn Foods Pty, 3 Ricky Way, Epping, Victoria, Australia; c Mass Spectrometry and Proteomics Facility, The Bio21 Molecular Science and Biotechnology Institute, The University of Melbourne, Melbourne, Victoria, Australia; d Griffith Institute for Drug Discovery, Griffith University, Nathan, Queensland, Australia

**Keywords:** Biotechnology, Microbiology, Food engineering, Probiotics, Antimicrobial, Microbial biotechnology, Proteomics, Peptides, *Lactobacillus*, Bacteriocin, Genomics, Circular, Cyclic, Protein cyclisation, Probiotic, Food microbiology, Natural product

## Abstract

*Lactiplantibacillus plantarum* B21 isolated from Vietnamese sausage (*nem chua*) has previously displayed broad antimicrobial activity against Gram-positive bacteria including foodborne pathogens *Listeria monocytogenes* and *Clostridium perfringens*. This study successfully identified the antimicrobial agent as plantacyclin B21AG, a 5668 Da circular bacteriocin demonstrating high thermostability, resistance to a wide range of pH, proteolytic resistance and temporal stability. We report a reverse genetics approach to identify and characterise plantacyclin B21AG from first principles. The bacteriocin was purified from culture supernatant by a three-step process consisting of concentration, n-butanol extraction and cation exchange chromatography. A *de novo* peptide sequencing using LC-MS/MS techniques identified two putative peptide fragments which were mapped to the genome sequence of *L. plantarum* B21. This revealed an ORF corresponding to a putative circular bacteriocin with a 33-amino acid leader peptide and a 58-amino acid mature peptide encoded on a native plasmid pB21AG01. The bacteriocin is shown to be a small cationic predominantly α-helical protein (69%). The corresponding gene cluster, consisted of seven genes associated with post-translational circularisation, immunity and secretion. Whilst plantacyclin B21AG is 86% identical to the newly published plantaricyclin A it is more highly cationic having a net charge of +3 due to an additional basic residue in the putative membrane interaction region. This and other substitutions may well go some way to explaining functional differences. The robust nature of plantacyclin B21AG, its antimicrobial activity and associated machinery for cyclisation make it an interesting biotechnological target for development, both as a food-safe antimicrobial or potentially a platform technology for recombinant protein circularisation.

## 1 Introduction

Food borne disease due to pathogen contamination is a major concern in the food industry [1]. Reports estimate that 4.1 million foodborne gastroenteritis cases occur in Australia annually, costing the country about $1.2 billion per year [2, 3]. Discovery of antimicrobial agents which contribute to food safety will have a growing economic and social significance and are a high priority for the food industry. There are concerns with the use of chemical food additives and preservatives [4], with a demand for alternative approaches to food safety, including natural products to control the growth of food spoilage bacteria and food-borne pathogens [5].

Lactic acid bacteria (LAB) have long been associated with food and feed fermentation, specifically as a starter culture in the production of fermented meat, vegetables, fruit, alcoholic beverages, dairy products and silage [6, 7]. They have been shown to inhibit the growth of food spoilage bacteria as well as a beneficial influence on the nutritional, organoleptic and shelf-life characteristics of fermented food products [8, 9, 10]. The preservative effect of LAB is due to the production of antimicrobial substances, including organic acids, hydrogen peroxide, diacetyl, bacteriocins and bacteriocin-like antimicrobial substances [10, 11]. Among these antimicrobial components, bacteriocins have received particular attention in recent years because of their application in the food industry as natural preservatives, and as potential antimicrobial peptides or antibiotic-like molecules targeting multi-drug resistance pathogens [5, 12].

Over the past few decades, many bacteriocins have been identified and studied extensively in LAB [13]. Several approaches have been taken to classify bacteriocins. The original classification of bacteriocins from LAB was suggested by Klaenhammer [14] based on the biochemical and genetic properties. Circular bacteriocins are distinguished from other classes of bacteriocins as they go through a unique post-translational modification, specifically an N to *C termini* ligation through an amide bond. Sequence and phylogenetic analysis have demonstrated two distinct families of circular bacteriocins, i and ii [15]. Family i circular bacteriocins tend to be more cationic and have higher isoelectric points (pI > 9), including enterocin AS-48 [16], enterocin NKR-5-3B [17], carnocyclin A [18], circularin A [19], garvicin ML [20], lactocyclicin Q [21], leucocyclicin Q [22], uberolysin [23], pumilarin [24] and most recently cerecyclin [25]. Family ii circular bacterocins are more anionic and have lower isoelectric points (pI < 7), including acidocin B [26], butyrivibriocin AR10 [27], gassericin A [28] and plantaricyclin A [29]. Circularisation contributes to some unique properties of these bacteriocins including high thermostability, resistance to a wide range of pH, proteolytic resistance and temporal stability, making them an interesting target for commercial applications [30].

Several *Lactiplantibacillus*/*Lactobacillus* spp. were previously isolated from Vietnamese fermented sausage, *nem chua* [31]. Among these isolates, *L. plantarum* B21 demonstrated one of the strongest antimicrobial activities against a range of bacterial strains due to a combination of acid and bacteriocin production [32]. In this study, we have purified and characterised a new bacteriocin from *L. plantarum* B21 using a first principles reverse genetics approach. Comparison with recent genome data has allowed a full description of the gene cluster encoding proteins associated with the post translational circularisation, production and secretion of the bacteriocin in addition to genes associated with self-immunity.

## 2 Results and discussion

### 2.1 Antimicrobial spectrum of *L. plantarum* B21

The cell free supernatant (CFS) from *L. plantarum* B21 demonstrated strong antimicrobial activity against closely related species and strains, such as *L. plantarum* A6, *L. plantarum* ATCC 8014 and *L. arabinosus* 17-5. In later work we demonstrated that only a single bacteriocin is present and by way of calibration, a strong positive result for a sensitive strain such as *L. plantarum* A6 corresponds to a MIC (minimum inhibitory concentration) value of 0.156 μM measured with pure bacteriocin. The MBC (minimum bactericidal concentration) value for this strain, was 2.5 μM, some 16 x higher than MIC. CFS also showed strong antimicrobial activity against one other LAB's tested in this study, *Lactococcus lactis* 345-18, but it is only weakly active against *L. brevis* 19012 (Table 1). However, there was evidence of some antimicrobial activity against foodborne pathogen strains, *Clostridium perfringens* 52/6-1 and *Listeria monocytogenes* 192/1-2 ACM 3173 strains (single strain examples in Table 1). A wider range of pathogenic strains should be tested in order to better understand the potential the antimicrobial spectrum of *L. plantarum* B21. In contrast, it was not shown to have any activity against any *E. coli* strains tested in this study. Other studies have reported that treatment with EDTA and lysozyme can render Gram-negative bacteria susceptible to LAB bacteriocins [13, 33, 34].

**Table 1:** Evaluation of *Lactobacillus plantarum* B21 antimicrobial activity by WDA assay.

Indicator strain	Antimicrobial activity∗
*Lactobacillus plantarum* A6	++++
*Lactobacillus plantarum* ATCC 8014^a^	+++
*Lactobacillus arabinosus* 17-5^b^	++++
*Lactococcus lactis* 345-18^b^	+++
*Lactobacillus brevis* 19012^b^	+
*Lactobacillus casei* ATCC 7469^a^	-
*Staphylococcus aureus* ATCC 25923^a^	-
*Escherichia coli* ATCC 25922^a^	-
*Escherichia coli* ATCC 35218^a^	-
*Escherichia coli* NCTC 9001^c^	-
*Listeria monocytogenes* 192/1-2 ACM 3173^b^	+
*Clostridium perfringens* 52/6-1^b^	+

*Lactobacillus plantarum* A6 was isolated from nem chua [31].

∗ Positive inhibitory activity [+], No inhibitory activity [-].

a Strains obtained from American Type Culture Collection (ATCC).

b Strains obtained from RMIT University Culture Collection.

c Strain obtained from National Collection of Type Cultures (NCTC).

### 2.2 Sensitivity of *L. plantarum* B21 antimicrobial substance(s) to heat and proteolytic enzymes

**Figure 1:**
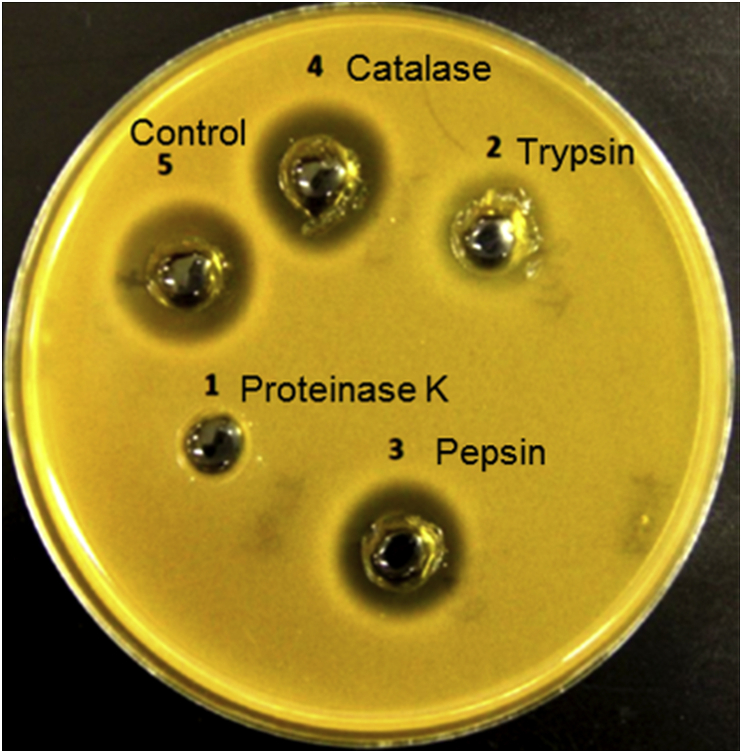
**Sensitivity of *Lactobacillus plantarum* B21 bacteriocin to proteolytic enzymes.** (1) Proteinase K; (2) Trypsin; (3) Pepsin; (4) Catalase; (5) Untreated cell free supernatant as control.

The initial putative antimicrobial substance(s) produced by this strain remained active after exposure to temperatures ranging from 40 °C to 80 °C for 20 min. No activity was observed after a 20 min heat-treatment at 100 °C. These results demonstrate a modest thermostability. The antimicrobial activity of *L. plantarum* B21 CFS was completely eliminated with proteinase K but it was significantly more resistant to trypsin and pepsin (Figure 1), strongly implying that the antimicrobial component was proteinaceous in nature. Resistance to pepsin and trypsin has been linked with circularisation [35]. Proteinase K is a broad-spectrum serine protease which predominantly cleaves the peptide bond adjacent to the carboxyl group of aliphatic and aromatic amino acids with blocked alpha amino groups [36]. Pepsin also has broad specificity with a preference for cleavage C-terminal to phenylalanine and leucine whereas trypsin cleaves peptides on the C-terminal side of basic residues such as lysine and arginine, all of which are abundant in the sequence [37, 38].

### 2.3 Production of *L. plantarum* B21 antimicrobial substance(s) across a wide range of pH

**Sup Figure 1:**
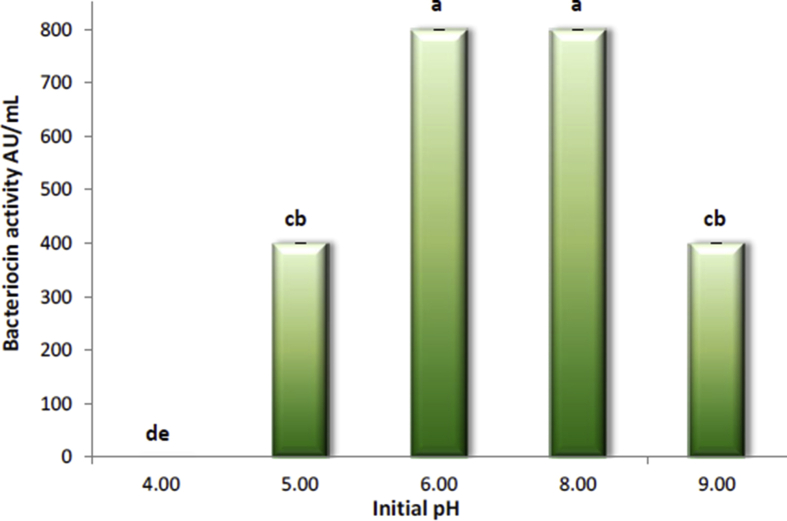
Graph demonstrating the effect of initial pH on bacteriocin production. ANOVA was used to determine the significance of differences at a confidence level of *p* < 0.05 as identified by different letters.

The effect of initial pH on the production of antimicrobial substance(s) was also tested. The antimicrobial substance(s) was optimally produced with an initial pH between 6 – 8, while a little less so at pH 5 and 9 (Sup Figure 1). No activity was detected in MRS broth with an initial pH of 4 while growth occurred. Our results are in accordance with several reports on bacteriocin production, where maximum production was observed between pH 5.5 to 7.5 whilst low levels were observed at more extreme pH, 5 and 9 [39, 40, 41, 42]. This data strongly imply that the antimicrobial substance is a secreted bacteriocin, which is produced across a wide range of pH values from 5 – 9.

### 2.4 Analysis of the *L. plantarum* B21 whole genome sequence

**Sup Figure 2:**
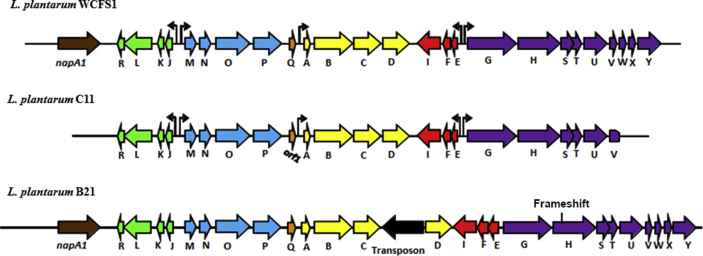
Genetic map comparison of the *pln* loci for *L. plantarum* B21, WCFS1 and C11. *L. plantarum* reference strains C11 and WCFS1 data obtained from previously publish works [47, 54, 92]. Small black arrows represent promoter sequences. The *pln* loci represented by arrows in different colours corresponding to each operon, yellow: regulatory operon (*plnABCD*); red/green: two-peptide bacteriocins operon (*plnEFI* and *plnJKLR*); blue: *plnMNOP* operon, purple: bacteriocin transport operon (*plnGHSTUVWXY*), black: transposon.

A gapless genome was obtained for *L. plantarum* B21 (GenBank Accession No. CP010528) and subjected to bacteriocin mining analyses. BAGEL3 detected several loci (*PlnK*, *PlnJ*, *PlnF*, *PlnN*, *PlnA* and *PlnE*) associated with the production of a classical plantaricin, a two-peptide linear bacteriocin [43, 44]. Further genome annotation through RAST revealed the loci within this plantaricin (*pln*) gene cluster to be chromosomally-encoded and strongly linked in an associated operon. The genetic organisation of the *pln* loci found in *L. plantarum* B21 is very similar to that of *L. plantarum* C11 and *L. plantarum* WCFS1 (Sup Figure 2) [45, 46, 47]. Five operons, i.e. the regulatory operon (*plnABCD*), the bacteriocin production and immunity operons (*plnJKLR*, *plnMNOP*, *plnEFI*) and the operon associated with bacteriocin transport (*plnGHSTUVW*) were detected. Interestingly however, in *L. plantarum* B21 a transposon (1,319 bp) was found to be located between the *plnC* and *plnD* gene (Sup Figure 2) and the insertion of this transposon is likely to impair the function of the *plnABCD* regulatory operon. An additional deletion and resultant frameshift were also identified at position 382 of *pln*H, the bacterocin ABC transporter. Both observations suggesting that the main *pln* locus had been impaired or substantially deactivated in this strain. Given that no other bacteriocin was detected in a search of the main genome sequence, a first principles reverse genetics approach was started in parallel to identify the bacteriocin while additional episomal plasmids were being characterised and sequenced [48].

### 2.5 Purification of the putative bacteriocin

**Figure 2:**
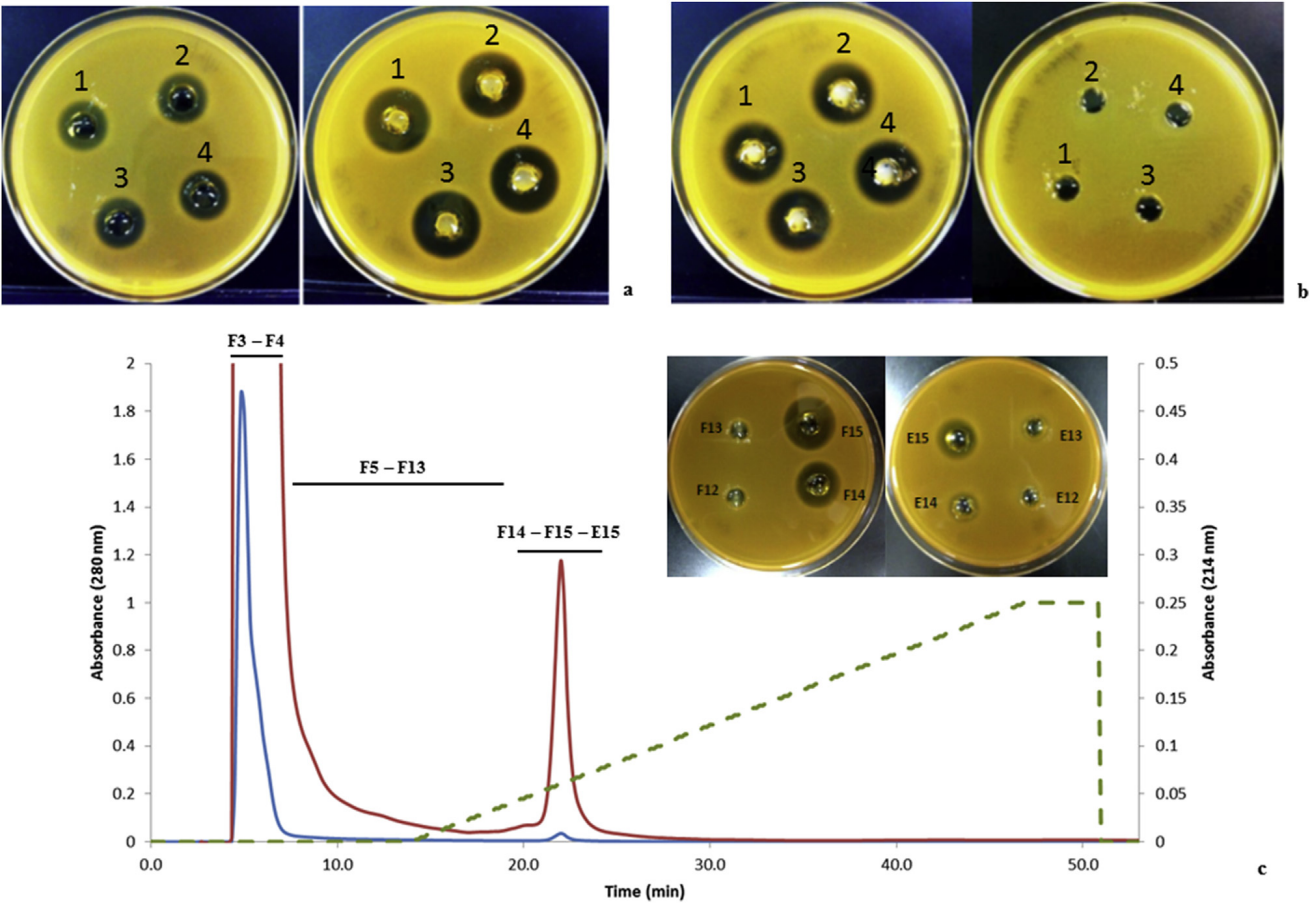
**Purification of bacteriocin from the CFS of *Lactobacillus plantarum* B21. *L. plantarum* A6 was used as indicator strain in all WDA assays.** (a) *L. plantarum* B21 CFS bacteriocin activity using WDA assay before (left) 800 AU/mL and after (right) at 12,800 AU/mL concentration through an ultrafiltration cell with 10 kDa membrane cut off. (b) The WDA assay examining the antimicrobial activity of organic (left) and aqueous phase (right) from the n-butanol extraction. Well 1–4 represent four technical replicates for each assay. (c) FPLC of the active fractions from the NAP10 gel filtration column. Eluted protein was detected at 280 nm (blue line) and 214 nm (red line). Fractions F14, F15 and E15 showed the strongest antimicrobial activities based on the WDA assay.

The bacteriocin was purified from the CFS of *L. plantarum* B21 through a size dependant ultrafiltration concentration, solvent extraction, and cation exchange chromatography. Well diffusion agar (WDA) analysis with serial dilution was performed after each step to monitor the antimicrobial peptide concentration. The starting bacteriocin activity was calculated as 800 (AU/mL) against *L. plantarum* A6. The use of an ultrafiltration system with a 10 kDa cut off membrane resulted in 16-fold concentration of the bacteriocin, which increased the inhibitory activity from 800 (AU/mL) to 12,800 (AU/mL) (Figure 2a), and no inhibitory activity was detected in the filtrate. The concentrated bacteriocin was extracted into n-butanol and the organic phase was dried under a nitrogen stream or freeze dried. The WDA assay of the dried n-butanol phase (resuspended in 20 mM sodium phosphate buffer, pH 6) showed strong bacteriocin activity (6,400 AU/mL) against *L. plantarum* A6 while no activity was observed in the aqueous phase, confirming that n-butanol extraction achieved 100% recovery of the bacteriocin activity (Figure 2b). The extraction and extent of recovery seemed to be even more efficient than reported for other bacteriocins where some residual activity is always observed in aqueous phase [49, 50]. Size exclusion on a G25 NAP 10 achieved buffer exchange and helped to remove excessive lipopolysaccharides prior to further chromatography. FPLC cation exchange chromatography (Uno S6, Biorad) resulted in a single bound peak in absorbance at 214 nm and a small peak at 280 nm corresponding to eluted protein (Figure 2c). The presence of one major peak was attributed to the selectivity of the n-butanol extraction, combined with the strongly basic nature of the bacteriocin, exhibiting an overall positive charge at pH 6.0. Three fractions taken across the single absorbance peak at 214 nm and 280 nm (F14, F15 and E15) showed the strongest bacteriocin activity (Figure 2c). Fractions F14 and F15 were pooled and concentrated. The amount of pure protein estimated by protein assay and calculated UV extinction coefficient were in good agreement. Purification and yield data for each stage of purification was used to back calculate the original concentration in the culture supernatant at approximately 1 mg/L. The amount was comparable to but perhaps a little less than that reported in the literature (~2.5 mg/L - 5.5 mg/L) [26, 50].

Interestingly, MALDI-TOF-MS identified the same bacteriocin MS peak in the flow through fraction, accounting for 20–30% of the active protein but as a component of a complex mixture. This may be either because of higher order aggregation/assembly or because of association with some other anionic contaminant, masking its charge properties. This may also reflect limited solubility in aqueous buffers. No attempts were made to salvage this protein by re-chromatography.

### 2.6 Stability and antimicrobial activity of the purified bacteriocin

**Sup Figure 3:**
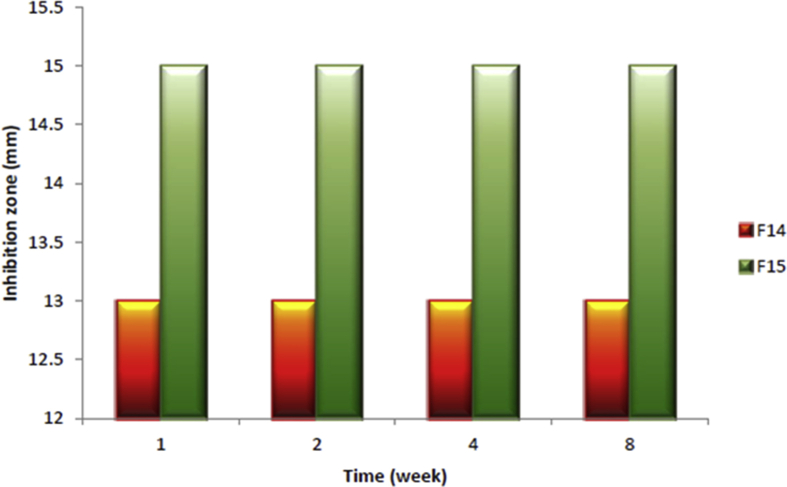
Graph showing the activity of plantacyclin B21AG after storage at 4°C for eight weeks. Green bars show the F14 fraction from FPLC while orange bars show the F15 fraction.

The purified bacteriocin (F14 and F15) was stored at 4 °C and examined for bacteriocin activity over an eight weeks period (Sup Figure 3). The bacteriocin remained 100% active against *L. plantarum* A6 and showed no reduction in activity over the period. This bacteriocin displayed high antibacterial potency against *L. plantarum* A6, with a MIC value of 0.156 μM. The MBC value of the bacteriocin was 2.5 μM, which is 16 times higher than the MIC value. This excellent stability is in complete contrast to that reported for classical linear two component bacteriocins [51, 52, 53], again suggesting that the antimicrobial substance produced by *L. plantarum* B21 could be a more stable circular bacteriocin.

### 2.7 Molecular weight and purity analysis

**Figure 3:**
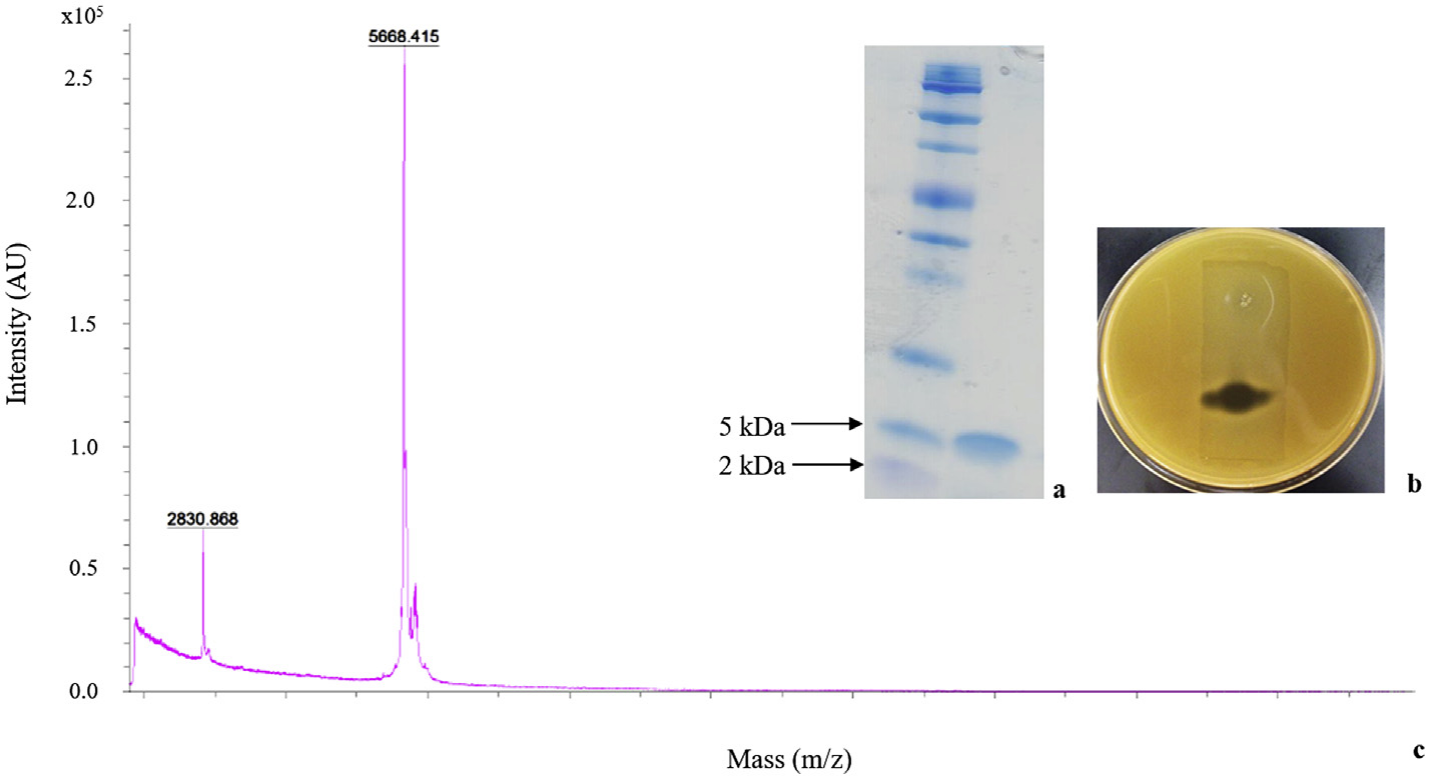
**Molecular weight and purity determination by Tris-Tricine-SDS-PAGE gel and MALDI-TOF-MS analysis.** (a) SDS-PAGE gel image; lane 1: Low molecular protein marker; lane 2: *L. plantarum* B21 purified bacteriocin. (b) Antimicrobial assay of the SDS-PAGE gel overlayed with the indicator *Lactobacillus plantarum* A6. (c) MALDI-TOF-MS spectrum of the purified *L. plantarum* B21 bacteriocin showing the singly (5668 Da) and doubly (2830 Da) charged species. Full gel picture found in Sup Figure. 5.

The purified bacteriocin showed a single band on a Tris-Tricine-SDS-PAGE gel (Figure 3a), corresponding to approximately 5 kDa, most bacteriocins are reported to be low molecular weight proteins of less than 10 kDa [54, 55, 56, 57, 58, 59]. After washing extensively in sodium acetate buffer overnight and overlay onto an agar plate with a susceptible indicator strain an inhibitory zone was observed around the protein band on the second half of the gel, confirming that some renatured activity is present and the single protein band is responsible for bacteriocin activity (Figure 3b). A single major peak was observed for the peak fraction by MALDI-TOF MS at 5668 Da (Figure 3c) and a second small peak (2830 Da) appeared to be a 2 + species.

**Figure 4:**
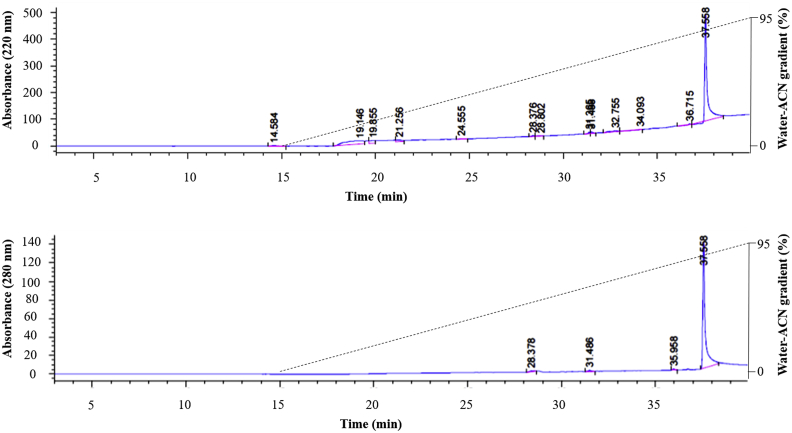
**RP-HPLC elution profile of the *L. plantarum* B21 FPLC purified bacteriocin.** Top figure A_220_; Bottom figure A_280_. An analytical ZORBAX Eclipse C_18_ column fitted to an Agilent 1100 RP-HPLC equipment was used with a flow rate of 1 mL/min. The bacteriocin was eluted with approximately 80 % water-ACN.

When the purified bacteriocin was injected onto a ZORBAX Eclipse analytical C_18_ column (4.6 × 150 mm), nothing was eluted in the 60% ACN-water gradient. When the ACN concentration gradient was increased to 95%, a single hydrophobic bacteriocin protein was eluted from the column at 37.5 min using a 40-min linear water-ACN gradient. This corresponded to the single 5668 Da bacteriocin (Figure 4). This experiment was repeated four times and the peaks from all runs overlapped. Failure to elute the bacteriocin protein from a C_18_ column using 60% water-ACN strongly suggested that the *L. plantarum* B21 bacteriocin protein molecule was more hydrophobic than other classical bacteriocins and consisted of a single polypeptide. SDS-PAGE electrophoresis, MALDI-TOFMS and RP-HPLC analyses all confirmed a very high level of purity and homogeneity of the FPLC-purified bacteriocin sample.

### 2.8 Bacteriocin peptide *de novo* sequencing and fragment analysis

**Figure 5:**
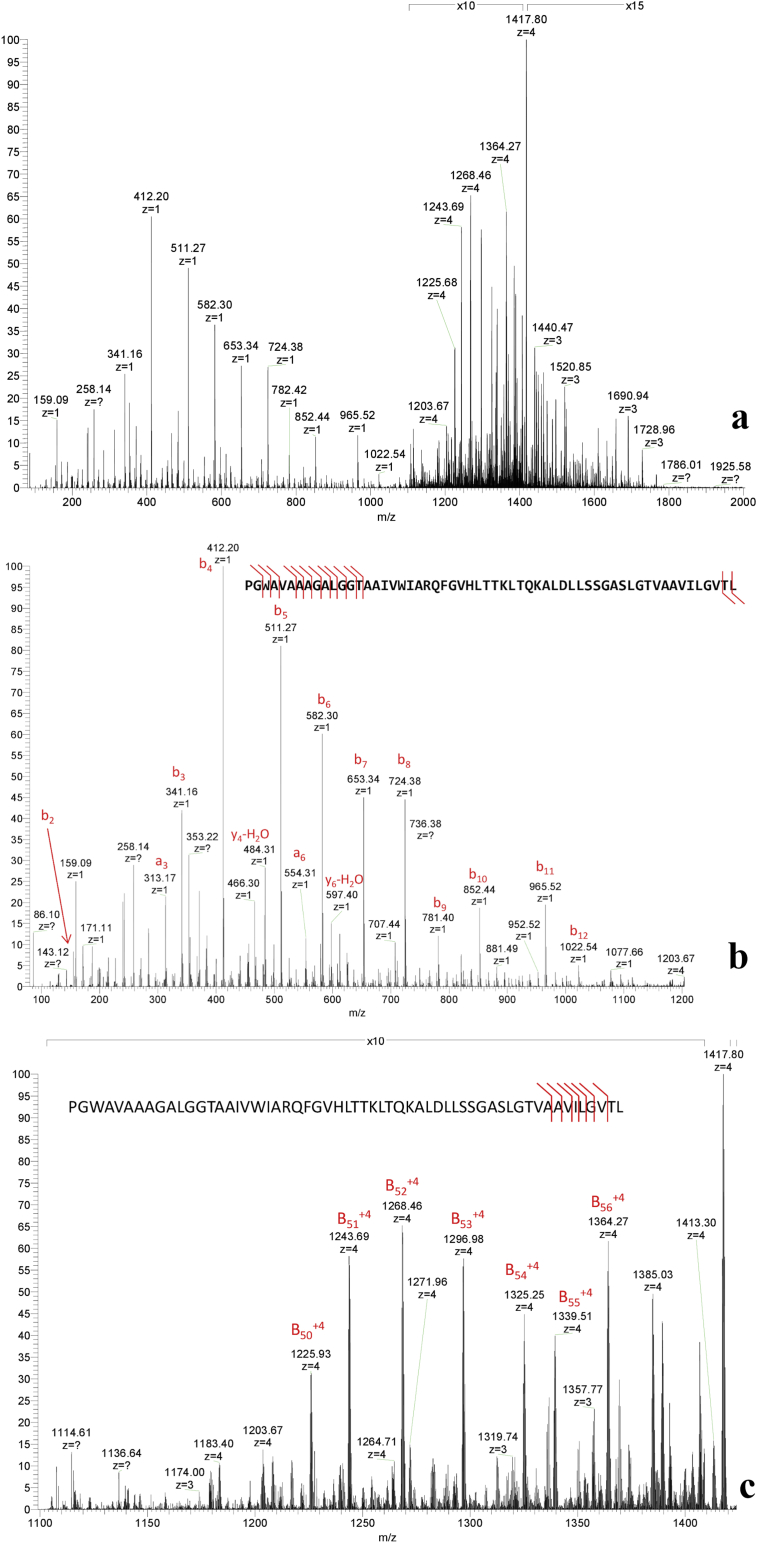
***De novo* peptide sequencing of the *L. plantarum* B21 bacteriocin using LC-MS/MS techniques.** (a) The +4 species centred at 1417.81 was manually selected for fragmentation analysis. (b) Identification of the b ions. Peptide sequence manually extracted from the MS/MS data was PGWAVAAAGALG. (c) Identification of the late b ions. Peptide sequence obtained was AAVILGV.

**Figure 6:**
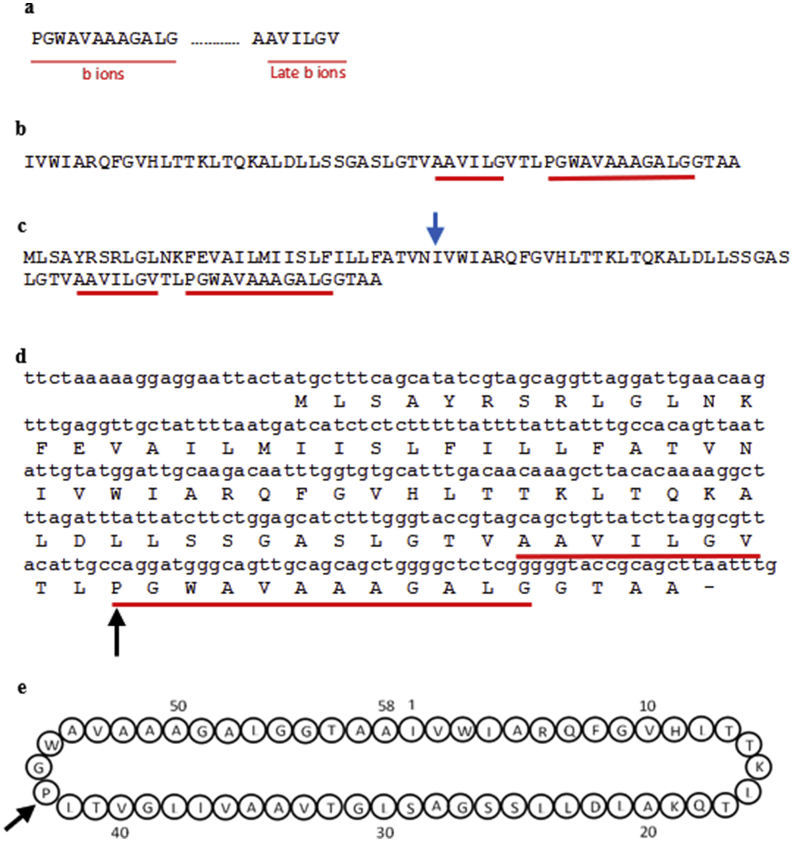
**Determination of the amino acid and nucleotide sequence of plantacyclin B21AG.** (a) Amino acid sequences obtained from *de novo* peptide sequencing using LC-MS/MS. (b) Plantacyclin B21AG mature peptide corresponding to the linear isotopic mass of 5682 Da, confirming the data obtained by MS/MS analysis. (c) Plantacyclin B21AG full putative sequence deduced from translation of the predicted ORF in pB21AG01. The hypothetical cleavage site of the leader peptide is indicated by a blue arrow. (d) The nucleotide sequence obtained from the *L. plantarum* B21 genome data corresponding to the plantacyclin B21AG amino acid sequence. The symbol (-) demonstrates the translation stop codon. (e) Predicted circular structure of plantacyclin B21AG. The black arrows show the main ion fragmentation site identified by the proteomic analysis.

Initial attempts to determine the N-terminal amino acid sequence of the purified *L. plantarum* B21 bacteriocin by Edman-degradation failed (data not shown), suggesting that the peptide may be N-terminally blocked. A *de novo* sequencing approach was then taken to identify the peptide sequence of the purified bacteriocin peptide using an ESI-LC-MS/MS technique. This identified a strong precursor ion at m/z 1417.0630 (monoisotopic peak) which corresponded to an intact monoisotopic mass of 5664.252 (Figure 5a). This was consistent with the previous mass measurements from MALDI-TOF MS and Tris-Tricine-SDS-PAGE. To improve the MS/MS data, a targeted run was setup in which the m/z 1417 precursor (with an isolation width of 4 Da) was subject to both CID and HCD at three different energy levels and activation Q of 0.25 (CID) or activation time of 0.1ms (HCD). The spectrum produced from HCD failed to identify any candidate sequence in the existing MASCOT databases, so the obtained sequence tags were extracted manually (PGWAVAAAGALG and AAVILGV, Figure 5b, c) and BLAST was used to search a six-frame translation of the *L. plantarum* B21 genome including plasmid DNA. These amino acid peptide tags corresponded to an open reading frame on a 20 kb contig, which did not form part of the main chromosome. This result suggests that the putative bacteriocin is encoded on a 20kb native plasmid which was subsequently named as pB21AG01 (GenBank Accession No. CP025732). A putative bacteriocin mature protein sequence was obtained from the predicted ORF (Figure 5b). From the mass spectrum data it appeared that the N-terminal sequence of the peptide started with the sequence PGWAVAAAGALG (Figure 5b) and the AAVILGV (Figure 5c) sequence was close to the C-Terminus; however comparison to the predicted amino acid from the genome data, indicated that AAVILGV sequence occurs just before the PGWAVAAAGALG sequence in the predicted ORF and was not separated by more than ~4000 Da of mass (Figure 6). The precursor mass of the peptide was found to be 18 Da less than the apparent mass of the predicted mature amino acid sequence. This strongly implies that protein is cyclic and is therefore a circular bacteriocin. The formation of a peptide bond between the N and C-Terminus and resulting loss of water resulted in mass discrepancy of 18 Da between the gene sequence (5682 Da) and the experimental mass of 5664 Da. After the full putative peptide sequence was deduced from the genome data, the remainder of the peptide sequence could be traced and confirmed in the MS/MS data (Figure 5b, c) and the *L. plantarum* B21 circular bacteriocin peptide was named plantacyclin B21AG. It is one of only a relatively few to be experimentally confirmed in this way at the protein level.

The behaviour of plantacyclin B21AG during the purification is fully consistent with the properties of a circular bacteriocin. Firstly, plantacyclin B21AG could not be sequenced by N-terminal sequencing, similar to other circular bacteriocins, including enterocin NKR-5-3B [17], lactocyclicin Q [21] and butyrivibriocin AR10 [27]. Secondly, plantacyclin B21AG showed some level of resistance to standard proteolytic enzymes for which cleavage sites were subsequently shown to exist. It has been reported that circular bacteriocins are generally more stable than classical bacteriocins [59, 60, 61, 62]. They are less susceptible to digestion by endoproteinases and this probably increases their spectrum of activity [20, 21]. Maqueda et al. [59] suggested that substantial part of the increased stability of enterocin AS-48 was due to the entropic constraints induced by the cyclic nature of the polypeptide chain. Plantacyclin B21AG was also shown to behave very hydrophobically on RP-HPLC column, requiring a high concentration (~80%) of ACN for elution. This behaviour has been observed for other circular bacteriocins like enterocin AS-48 [59]. Finally, the protein sequence of the ORF corresponding to plantacyclin B21AG was shown to have similarity to other known circular bacteriocins.

### 2.9 Protein sequence analysis of plantacyclin B21AG

The 20 kb pB21AG01 which contains the structural gene of plantacyclin B21AG was subjected to comparative analysis with BAGEL3. Two strong similarity hits to circular bacteriocins were found, gassericin A (52.6% match, E-value = 2e-21) and pentocin (58.2% match, E-value = 1e-25), essentially confirming the result of *de novo* MS sequencing. The putative bacteriocin peptide has 91 amino acids (pre-peptide) consisting of a 33-amino-acid leader peptide and a 58-amino-acid pro-peptide (Figure 6d). The putative cleavage site for removing the leader peptide is located between asparagine and isoleucine and has homology to the leader peptide cleavage site in gassericin A and acidocin B [26, 28]. The 58-amino-acid peptide is predicted to undergo a post-translational modification that results in the linking of the N-terminal asparagine to the C-terminal isoleucine, with the elimination of a water molecule, resulting in the active and mature B21AG molecule that is secreted. The leader peptides in circular bacteriocins can vary in length, from 2 to more than 30 amino acids [18, 20, 35, 63, 64]. BLASTp analysis of the mature bacteriocin peptide (58 amino acids) showed 86% and 67% identity to the circular bacteriocin plantaricyclin A [29] produced by *L. plantarum* NI326 (accession number WP_053266997.1) and predicted circular bacteriocin pentocin KCA1 (accession number EIW14922), respectively. It also showed 65% identity to known circular bacteriocins produced by other *Lactiplantibacillus*/*Lactobacillus* species, including gassericin A [63] and acidocin B [26] (accession numbers WP_012621083.1 and CAA84399.1). These results demonstrate that plantacyclin B21AG is a new member of the circular bacteriocin class produced by *Lactobacillus* spp. with high similarity to plantaricyclin A (86% identity), the circular bacteriocin produced by *L. plantarum* NI326 [29].

**Sup Figure 4:**
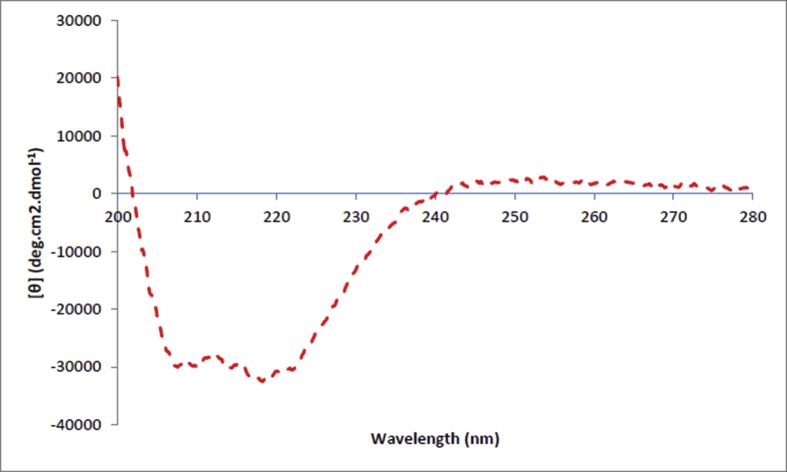
CD spectra of purified plantacyclin B21AG in 20 mM sodium phosphatebuffer. Spectra were recorded in a Jasco J-815 CD spectrometer in 1 cm cell at 5.3 μMprotein concentration with a scan speed of 50 nm/min and temperature control at 25°C. Spectra were average from three scans.

The amino acid composition of the mature circular bacteriocin consists of a very high proportion (59%) of hydrophobic amino acid residues (Ala, Val, Leu, Ile, Phe, Trp and Pro) and also uncharged hydrophilic amino acid residues (32%) (Gly, Ser, Thr and Gln). There is also high ratio of basic (Lys, Arg and His) relative to acidic amino acids (Asp) confirming a strong basic protein character. It has been hypothesised that basic residues such as Lys present a highly localised positive charge on the surface of the circular bacteriocins structure which is responsible for attracting the peptide to the surface of the negatively charged membrane [29, 64]. Unlike cyclotides, the cyclic peptides found in plants which contains disulfide bridges between cysteine residues for structural stability [65], plantacyclin B21AG does not contain any cysteine residues, suggesting that circularisation must contribute to their characteristic stability relative to their linear counterparts [35]. The bacteriocin peptide also contains three aromatic residues (two Trp and one Phe) and is able to renature in aqueous buffer following butanol extraction. The CD spectrum of plantacyclin B21AG (Sup Figure 4) is consistent with a structure which is 69% α helical. Such a high proportion of seemingly stable helical structure in such a small protein is quite unusual.

### 2.10 Genetic analysis of plantacyclin B21AG gene cluster

Plasmid annotation was performed using RAST to identify the operon associated with the production of plantacyclin B21AG. Seven open reading frames (ORF-A to ORF-G) were detected. The full amino acid sequence of *orfA* is 86% identical to *plcA*, a gene encoding for plantaricyclin A precursor (Table 2). The gene cluster of plantacyclin B21AG is found to be located on a 20kb native plasmid, pB21AG01 (GenBank Accession No. CP025732). We extracted the genomic data of plantaricyclin A and found that the bacteriocin gene cluster is located on a contig highly similar to pB21AG01 (91 % query cover, 92.3 % identity), suggesting plantaricyclin A is also plasmid encoded. There are some interesting differences between the antimicrobial activity of *L. plantarum* B21 and *L. plantarum* NI326. *L. plantarum* B21 is active against the *L. monocytogenes* strain tested in this study, but *L. plantarum* NI326 shows no activity against any of the *Listeria* strains examined. *L. plantarum* B21 also possesses antimicrobial activity against closely related species such as *L. brevis* and other *L. plantarum* strains but *L. plantarum* NI326 does not seem to [29]. Analysis of sequence alignments between these two closely related bacteriocins reveals only a small number of critical amino acid differences. The most distinct being a polar serine residue at position 15 for plantaricyclin A, whereas a basic lysine residue for plantacyclin B21AG. This is a significant difference and imparts an overall net charge of +3 for plantacyclin B21AG compared to +2 for plantaricyclin A. Positively charged residues have been shown to play an important role in the initial electrostatic interaction between the circular bacteriocin and the negatively charged phospholipid bilayer of the bacterial membrane [35]. This may be a plausible reason for differences in the antimicrobial spectrum observed between the two variants. It may be possible to assess the sensitivity of different indicator species/strains to plantacyclin B21AG using a putative docking molecule, such as the maltose ABC transporter, as in the case of garvicin ML [66]. Clearly, a high-resolution structure and a model for pore assembly and membrane penetration are required.

**Table 2:** Gene cluster putatively involved in the biosynthesis and self-immunity of plantacyclin B21AG.

ORF	Amino acid (length)	Best homolog, GenBank accession no. [organism]	% identity	Hypothetical function
*orfA*	91	Plantaricyclin A precursor, PlcA, PCL98053.1 [*Lactobacillus plantarum*]	88	Bacteriocin biosynthesis
*orfB*	157	Plantaricyclin A immunity protein, PlcD, PCL98052.1 [*Lactobacillus plantarum*]	94	Bacteriocin self-immunity
*orfC*	54	Plantaricyclin A immunity protein, PlcI, PCL98051.1 [*Lactobacillus plantarum*]	89	Bacteriocin self-immunity
*orfD*	227	Plantaricyclin A ATP-binding protein, PlcT, PCL98050.1 [*Lactobacillus plantarum*]	95	ABC-type multidrug transport system
*orfE*	214	Plantaricyclin A membrane transporter, PlcE, PCL98049.1 [*Lactobacillus plantarum*]	97	ABC-2 family transporter
*orfF*	173	Plantaricyclin A related protein, PlcB, PCL98048.1 [*Lactobacillus plantarum*]	90	Unknown
*orfG*	56	Plantaricyclin A related protein, PlcC, PCL98047.1 [*Lactobacillus plantarum*]	95	Unknown

**Figure 7:**
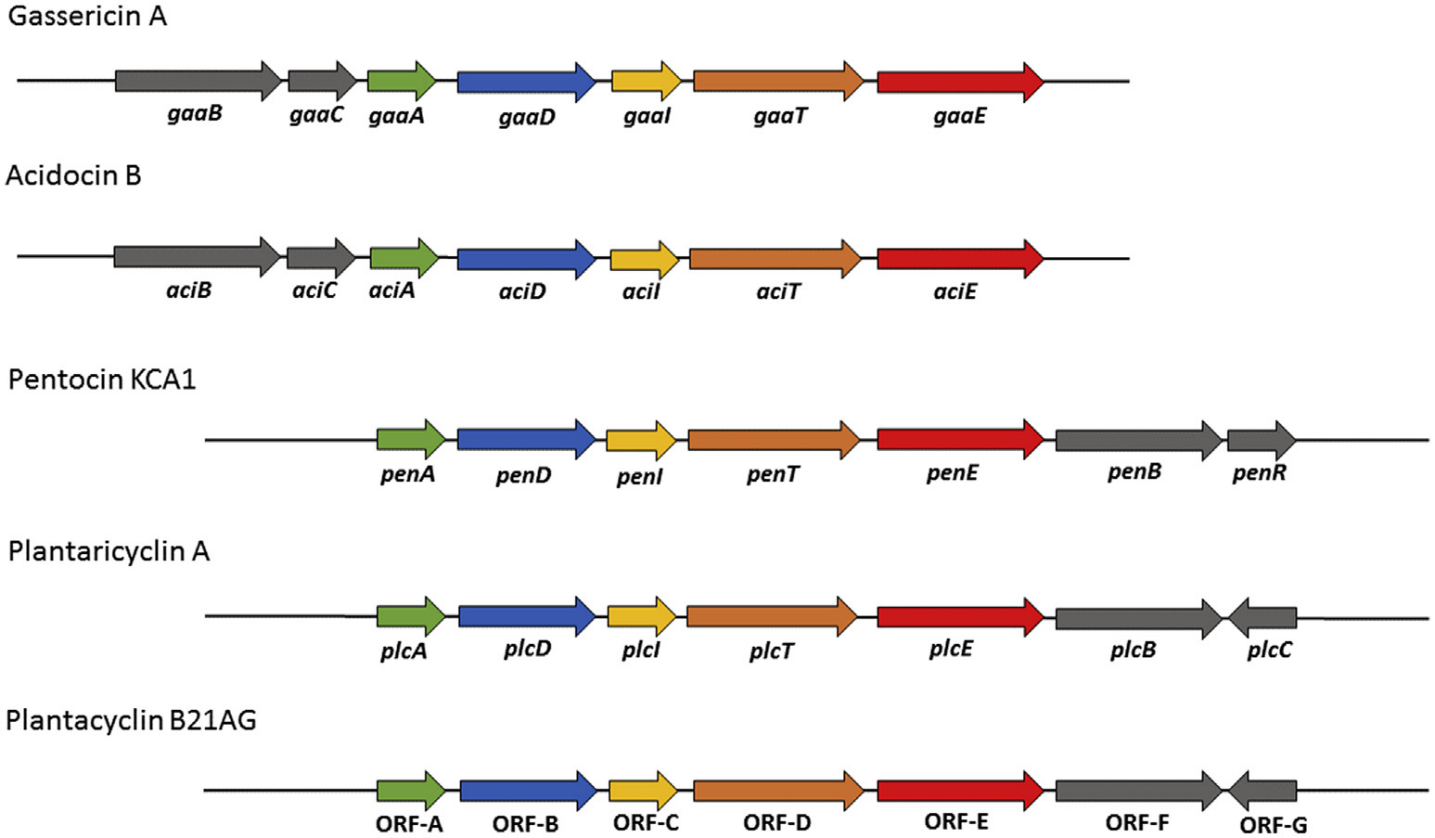
**Schematic representation of the gene clusters involved in the production of circular bacteriocin gassericin A, acidocin B, pentocin KCA1, plantaricyclin A and plantacyclin B21AG.** The genes are colour coded according to their known or putative functions. Green: Bacteriocin precursor; Blue: stage II sporulation protein M; Yellow: Immunity protein; Orange: ABC transporter; Red: Membrane transporter; Grey: Unknown protein.

Apart from the structural gene (*orfA*) responsible for bacteriocin production, we also identified six additional ORFs (Figure 7, *orfB* to *orfG*) that appeared to be involved in bacteriocin production and immunity within the bacteriocin gene cluster (Figure 7). The genetic organisation of plantacyclin B21AG mirrors that of plantaricyclin A. BLASTp analysis of the ORF-B putative protein sequence (157 amino acids) detected a stage II sporulation protein M (WP_158524160.1) (formerly known as the putative conserved domain from the DUF95 superfamily) [67], that shows 94% sequence identity to *plcD*, a gene encoding the plantaricyclin A immunity protein [29] (Table 2). It also shows some homology to other known circular bacteriocins, i.e 39%, 34%, 33% and 30% sequence identity to *PenD* from *L. pentosus* KCA1 [68], *AciD* from *L. acidophilus* M46 [26], *GaaD* from *L. gasseri* LA39 [69] and *BviE* from *Butyrivibrio fibrisolvens* AR10 [27], respectively. *AciD* is also a stage II sporulation protein M [26]. It has been demonstrated to be possibly involved in the biosynthesis of circular bacteriocin, as an immunity-associated transporter and as a secretion-aiding agent [70]. Kalmokoff [27], suggested that the *bviE* gene is involved in self-immunity to the circular bacteriocin, butyrivibriocin AR10. The dedicated immunity gene always appears to be located within the bacteriocin cluster [71]. The analysis of ORF-C putative protein sequence (54 amino acids) showed 89% identity to *plcI*, a plantaricyclin A putative immunity protein from *L. plantarum* NI326 [29] (Table 2). It also shares 38% sequence identity with putative immunity proteins *GaaI* from *L. gasseri* LA39 [69,72] *and aciI* from *L. acidophilus* M46 [26]. The presence of the putative immunity genes prevents self-killing of the bacteriocin producer, and it has also been shown in the case of plantaricyclin A that both immunity genes (*plcD* and *plcI*) are required for full immunity [29].

The ORF-D putative protein sequence (220 amino acids) shares 95% sequence identity to *plcT,* a gene that encodes plantaricyclin A ATP-binding ABC transporter protein [29] (Table 2). It is also 49% identical to *penT* from *L. pentosus* KCA1 [68], and 46% identical to both *aciT* from *L. acidophilus* M46 [26] and *gaaT* from *L. gasseri* LA39 [69], which is involved in the transportation of gassericin A. It has been reported that dedicated transmembrane translocators belonging to the ATP-binding cassette (ABC) transporter superfamily are involved in the cleavage of the leader-peptide from Class II bacteriocins and in the transportation of the mature bacteriocin molecule across the cytoplasmic membrane [71]. These results suggested that ORF-D gene appears to be involved in the cleavage of the leader peptide and transport of plantacyclin B21AG bacteriocin protein. Similarly, ORF-E putative protein sequence (214 amino acids) showed 94% sequence identity to *plcE*, a gene that encodes plantaricyclin A membrane transporter [29] (Table 2). It also showed 39%, 38% and 37% sequence identity to *penE* [68], *gaaE* [69] and *aciE* [26], respectively, encoding an ABC-2 transporter permease.

ORF-F putative protein sequence (173 amino acids) showed 90% sequence identity to *plcB*, which is a gene encoding for plantaricyclin A-related protein [29] (Table 2). It is 31% and 30% identical to *penB* [68], and *aciB* [26], respectively. It also shares 30% sequence identity to *gaaB* [69], a membrane protein with 5 predicted transmembrane segments (TMS) from *L. gasseri* which is involved in production of the circular bacteriocin, gassericin A. Similarly, ORF-G showed 90% sequence identity to *plcC*, a gene that encodes plantaricyclin A related protein [29] (Table 2). It also shares 30% sequence identity to *gaaC* [69] and *aciC* [26].

### 2.11 Comparisons to other circular bacteriocins

**Figure 8:**
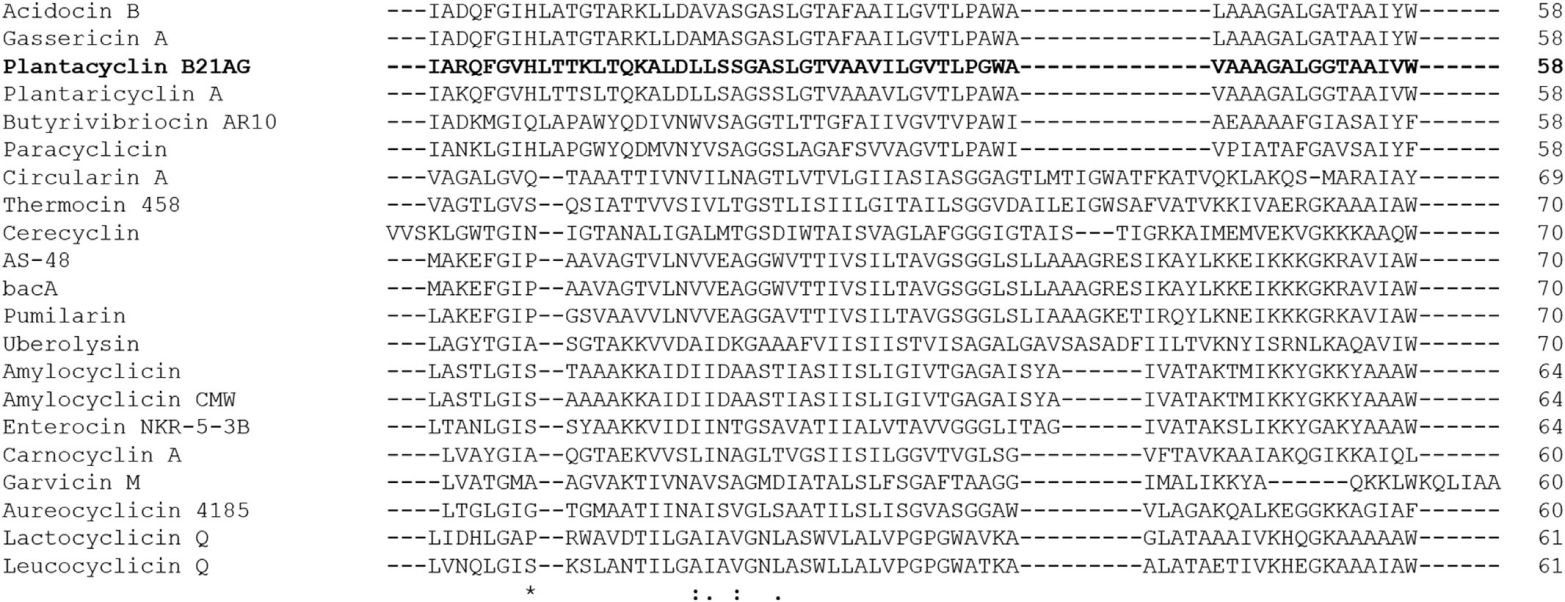
**Amino acid sequence alignment of circular bacteriocins.** Arrow indicates cleavage between leader peptide and mature peptide. Asterisks indicate fully conserved residues. Colons and periods indicate conservation between groups with strongly and weakly similar properties, respectively. Basic residues are highlighted in dark grey whereas acidic residues are coloured in light grey. Acn: Amylocyclicin, NKR-5-3B: Enterocin NKR-5-3B, CclA: Carnocyclin A, GarML: Garvicin ML, AclA: Aureocyclicin 4185, LycQ: Lactocyclicin Q, LcyQ: Leucocyclicin Q, Thermocin: Thermocin 458, UblA: Uberolysin A, AS-48: Enterocin AS-48, CirA: Circularin A, BviA: Butyrivibriocin A, GaaA: Gassericin A, AciB: Acidocin B, PlcA: Plantaricyclin A, B21AG: Plantacyclin B21AG.

**Figure 9:**
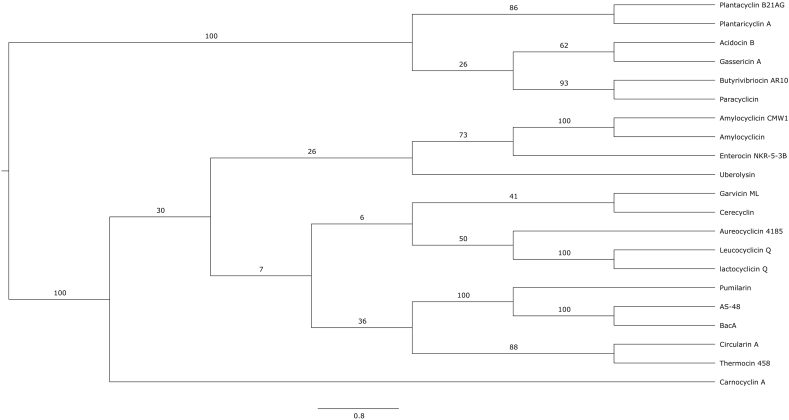
**Phylogeny of the mature sequences of all known circular bacteriocins.** The bacteriocins cluster into two main groups. Plantacyclin B21AG clusters within subgroup ii. Subgroup i contains the most variation of circular bacteriocins. Bootstrap values are shown. Asterisk shows plantacyclin B21AG. Tree based on amino acid sequence alignment without signal sequences. The sequences of family ii were permuted by 3 residues to better align with family i, built in RAxML then visualised in FigTree.

Multiple sequence alignment of all experimentally verified circular bacteriocins (Figure 8) revealed several interesting characteristics. As circular bacteriocins have no true N or *C terminus*, they can in principle be aligned at any starting point. Therefore, alignments were performed against the mature sequences, permuted by 3 residues (Sup Data 1) to improve the alignment between the two families. Family i circular bacteriocins have very low relative sequence homology. In contrast, family ii circular bacteriocins demonstrate much higher sequence homology and a similar length, with the exception of Butyrivibriocin AR10 (Figure 8) [35]. Most of the highly conserved residues of family ii members are hydrophobic amino acids (i.e. isoleucine, alanine, leucine, glycine, valine, proline and tryptophan), indicating the importance of hydrophobicity in the mode of action of these circular peptides, such as membrane pore formation [35]. Several basic residues (lysine and histidine) are also conserved among the circular bacteriocins (Figure 8), suggesting the positively-charged surface of the molecules plays a role in the initial attraction and subsequent insertion into the negatively-charged phospholipid bilayer of the target cell membrane [30]. Compared to other circular bacteriocins in subgroup ii, plantacyclin B21AG has a higher positive net charge of +3. The linear form of plantacyclin B21AG has a pI of 10 according to the ExPaSy ProtParam Tool, which is much higher than other circular bacteriocins in family ii [35]. This data suggests that the criteria for subgrouping of the circular bacteriocins should be revised. All circular bacteriocins in family ii are known to be plasmid-encoded except Butyrivibriocin AR10 [73, 74]. Phylogenetic analysis confirmed the two main groups of circular bacteriocins as previously described (Figure 9) [30]. Plantacyclin B21AG sits within family ii and is closely related to plantaricyclin A, followed by gassericin A and acidocin B. It is distantly related to butyrivibriocin AR10. Plantacyclin B21AG, plantaricyclin A, gassericin A and acidocin B contain seven genes in their gene clusters, i.e. (i) a gene encoding the mature peptide, (ii) a gene encoding an immunity protein, (iii) genes encoding a membrane transporter and a ATP-binding ABC transporter protein, (iv) a gene encoding the stage II sporulation protein M, and (v) genes encoding two unknown proteins [35]. The order of the genes is similar between plantacyclin B21AG and plantaricyclin A, thus they are more closely related to each other. The same for gassericin A and acidocin B. In contrast, the gene cluster of butyrivibriocin AR10 only contains five genes, lacking the two genes encoding for unknown proteins, thus more distantly related to the other bacteriocins in family ii.

## 3 Conclusions

An antimicrobial agent isolated from the supernatant of *L. plantarum* B21 was identified as a circular bacteriocin plantacyclin B21AG, the sole antimicrobial compound, the classical *pln* locus in this strain being substantially disabled It has a mass of 5668 Da, confirmed by both MALDI-TOF MS and *de novo* sequencing using ESI-LC-MS/MS. The purification process demonstrated a yield of approximately 19% and an overall purification factor of more than 8,000-fold. Back calculation from the purification table (Sup Table 1) suggested *L. plantarum* B21 produces approximately 1 mg/L bacteriocin in its CFS. The partially-purified plantacyclin B21AG was temporally stable, resistant to endopeptidases and produced in the pH range from 5 – 9. It had remarkable solubility in butanol and renatured spontaneously to a functional form in aqueous buffer. Its spectrum of antimicrobial activity included activity against both closely related *Lactobacillus* species, as well as foodborne pathogens such as *Clostridium perfringens* and *Listeria monocytogenes.* This newly identified circular bacteriocin while similar to plantaricyclin A has some additional features that may make it be desirable for food preservation as well as an important vehicle for structure function analysis.

## 4 Materials and methods

### 4.1 Bacterial strains and culture conditions

The bacteriocin-producing organism, *L. plantarum* B21 [74] and the indicator strain, *L. plantarum* A6 [75] were isolated from *nem chua*. The remaining indicator strains were either purchased from the American Type Culture Collection (ATCC) or obtained from RMIT University Culture Collection, Discipline of Bioscience and Food Technology, School of Science, RMIT University West Campus, Plenty Road, Bundoora, VIC 3083, Australia [31] as listed in Table 1. All LAB strains were grown in MRS broth (Oxoid, UK) at 30 °C for 24 h without shaking. For growth of the LAB strains on solid media, MRS agar (Oxoid, UK) was used and the plates were incubated at 30 °C for 48 h. *C. perfringens* and *L. monocytogenes* were grown anaerobically in Brain-heart infusion (BHI) (Oxoid, UK) broth at 37 °C and 30 °C, respectively.

### 4.2 Antimicrobial activity assay

The antimicrobial activity of *L. plantarum* B21 was examined using a well diffusion assay (WDA) [76]. Briefly, *L. plantarum* B21 was recovered from -80 °C in 5 mL MRS broth and incubated at 30 °C for 24 h. A 2% (v/v) overnight culture was used to inoculate 10 mL MRS broth and incubated in the same condition. The cell free supernatant (CFS) was harvested by centrifugation at 5000 × g for 20 min at 4 °C and neutralised to pH 6.0–6.5. Semi-solid MRS medium supplemented with 0.8 % agar was mixed with approximately 10^6^ CFU/mL of the indicator strain and allowed to solidify. Wells of 8-mm in diameter were perforated and 100 μL of the CFS was placed into each well. The agar plates were held at 4 °C for 2 h, followed by incubation at 30 °C for 18–24 h. The antimicrobial activities of the CFS were determined by measuring the diameters of the inhibition zones. Inhibition was recorded as negative if no zone was observed around the agar well. For quantitative measurement, two-fold serial dilutions of the CFS were prepared using PBS and the antimicrobial activities were expressed as arbitrary units (AU/mL) as described previously [39], calculated as ab × 100, which “a” is the dilution factor and “b” the last dilution demonstrating an inhibition zone of at least 2 mm in diameter.

### 4.3 Sensitivity of *L. plantarum* B21 CFS to heat and proteolytic enzymes

The CFS of *L. plantarum* B21 was harvested by centrifugation at 5,000 × g for 20 min at 4 °C. The CFS was heated to 40 °C, 60 °C, 80 °C and 100 °C for 20 min. The CFS was also neutralised to pH 6.5 and treated separately with proteinase K, trypsin and pepsin (Sigma-Aldrich, USA) at a final concentration of 1 mg/mL. Untreated CFS was included as a control whereas CFS treated with catalase (Sigma-Aldrich, USA) was used as a negative control. This was followed by WDA assay to examine the loss of antimicrobial activity of the heat- and proteases-treated CFS using *L. plantarum* A6 as indicator strain. The diameters of the inhibition zones observed from the treated CFS were compared against those produced by untreated CFS or catalase-treated, used as positive and negative controls, respectively. The loss or reduction in the size of the inhibition zone was taken as an indication of the sensitivity of the antimicrobial activity to heat treatment and proteolytic digestion.

### 4.4 The effect of initial pH on the production of *L. plantarum* B21 antimicrobial substance(s)

The effect of pH on the production of *L. plantarum* B21 antimicrobial substance(s) was performed by adjusting the initial pH of MRS broth to pH 4, 5, 6, 8 and 9, followed by growing the culture for 24 h (2% v/v inoculum) at 30 °C. The antimicrobial production was expressed as killing activity (AU/mL) using WDA method as described above.

### 4.5 Bacteriocin screening through whole genome sequencing of *L. plantarum* B21

The genome of *L. plantarum* B21 was sequenced using Illumina HiSeq 2000 sequencing platform (BGI, China) to a final coverage of 164-fold. The *de novo* assembly was performed using SOAPdenovo software [77]. The SOAPdenovo software was used in combination with PCR approach to close the gaps and correct misassembles. The Glimmer version 3.0 program was used for gene prediction. Functional gene annotation was performed using the NCBI Prokaryotic Genome Annotation Pipeline. The rRNAmmer, tRNAscan and Rfam software [78, 79, 80] were used to predict rRNA, tRNA and sRNA, respectively. The gapless genome sequence of *L. plantarum* B21 was subjected to BAGEL3 [81] and Rapid Annotation using Subsystem Technology (RAST) [82] analyses for bacteriocin screening. The resulting bacteriocin associated open reading frames (ORFs) were manually annotated using BLASTp against the non-redundant protein database at NCBI [83].

### 4.6 Purification of bacteriocin

The *L. plantarum* B21 bacteriocin was purified from the CFS by a three-step protocol process consisting of concentration, n-butanol extraction and cation exchange chromatography by Fast Protein Liquid Chromatography (FPLC). All purification steps were carried out at room temperature. *L. plantarum* B21 was used as an inoculum 2% (v/v) into 165 mL of MRS broth and incubated at 30 °C for 24 h without shaking. The CFS was prepared as previously described. The CFS was concentrated to 70 mL through a 10 kDa polyethersulfone membrane discs (Generon, UK) using an Amicon UF cell (Model 8200, Millipore, USA). The concentrated CFS was subjected to n-butanol extraction according to Abo-Amer [49] with a few modifications. The concentrated *L. plantarum* B21 CFS was extracted twice in 1:1 volume of water saturated n-butanol and centrifuged at 10,000 × g for 10 min. The n-butanol fraction containing the bacteriocin was freeze dried (FDU-8612, Operon Co. Ltd, Korea) to remove the solvent. The dried n-butanol fraction was redissolved in 20 mM sodium phosphate buffer, pH 6.0. Both the n-butanol and aqueous fractions were examined for antimicrobial activity. The redissolved protein sample was desalted using a NAP10 desalting column pre-packed with Sephadex G-25 resin (NAP™-10 Columns, Sephadex™ G25, GE Healthcare Life Sciences, UK) and eluted in 20 mM sodium phosphate buffer (pH 6.0). Purification of the desalted bacteriocin protein was performed using an Uno S-6 prepacked monolith cation exchange column (12 × 55 mm, Bio-Rad, USA) on a FPLC system (BioLogic DuoFlow System, Bio-Rad, USA). The Uno S-6 column was equilibrated with 20 mM sodium phosphate buffer, pH 6.0 (buffer A) and the desalted bacteriocin protein was eluted from the column with a linear NaCl gradient (0–1 M) in buffer A. A total of 33 fractions of 3 mL were collected and assayed for the antimicrobial activity. The two FPLC-fractions showing the strongest bacteriocin activity (F14 and F15) were pooled together (6 mL) and concentrated/buffer exchanged using an Amicon® Ultra-4 Centrifugal Filter Units (3 kDa, Millipore, Ireland). The final volume of the purified bacteriocin protein fraction was reduced to 200 μL. The concentration of the bacteriocin was measured after each step of protein purification using Pierce™ BCA Protein Assay Kit (Thermo Fisher Scientific, USA) according to manufacturer's instruction.

### 4.7 Stability of the bacteriocin antimicrobial activity

Two FPLC-purified fractions (F14 and F15), were stored at 4 °C for a period of eight weeks. At specific time intervals, 1, 2, 4 and 8 weeks, samples were examined for bacteriocin activity by WDA using *L. plantarum* A6 as an indicator. MIC assay was also performed using a broth microdilution method as described previously [84]. Briefly, *L. plantarum* A6 culture was prepared to a final concentration of approximately 1 × 10^5^ CFU/mL. Bacteriocin was 2-fold serially diluted from 10 μM to 0.02 μM. 100 μL of bacteria culture was added to each well of a sterile 96-well plate with different concentrations of bacteriocins and incubated overnight at 30 °C. Broth without bacteriocin and indicator strain served as positive and negative controls, respectively. MIC was determined as the lowest concentration of the bacteriocin that prevented visible growth by visual inspection. MBC was determined after the MIC incubation period by spreading 100 μL from each well that did not show visible growth on MRS agar and incubated overnight at 30 °C. MBC was defined as the lowest concentration of bacteriocin that killed the indicator strain completely [84]. Experiments were performed in triplicates with two biological replicates.

### 4.8 Determination of molecular weight and purity

The molecular weight and purity of the FPLC-purified bacteriocin was determined using tris-tricine-sodium dodecyl sulfate-polyacrylamide gel electrophoresis (Tris-Tricine-SDS-PAGE, 16.5% resolving gel) as previously described [85]. After electrophoresis at 60 V for 30 min followed by 100 V for 1.5 h, half of the gel was stained using a Coomassie-based staining solution (Expedeon, UK), while the other half was fixed in a fixing solution containing 20% isopropanol and 10% acetic acid for the examination of antimicrobial activity as described previously [57]. *L. plantarum* A6 was used as indicator strain. Precision Plus Protein™ Dual Xtra Standards (Bio-Rad, USA) was used to estimate the size of the bacteriocin. The same sample was also investigated by matrix-assisted laser desorption/ionization time-of-flight mass spectrometry (MALDI-TOF MS) [86] using an Autoflex Speed MALDI-TOF instrument (Bruker, Germany) containing a 355-nm Smartbeam II laser for desorption and ionization. The acceleration and reflector voltages were 20 and 23.4 kV in pulsed ion extraction mode. A molecular mass gate of 450 Da improved the measurement by filtering out most matrix ions. To determine the purity of the sample, the bacteriocin was diluted 10-fold in 0.1% trifluoroacetic acid (TFA) and subjected to analytical reverse-phase high-performance liquid chromatography (RP-HPLC) [87] using an Agilent 1100 HPLC system fitted with a ZORBAX Eclipse XDB analytical C_18_ column (4.6 × 150 mm, 5 μm particle size, Agilent Technologies, USA). The bacteriocin protein was eluted using a 40-min linear water-ACN gradient (increasing the gradient to 95%).

### 4.9 Circular dichroism (CD) spectroscopy

The circular dichroism spectra of purified bacteriocin protein were recorded from 280 to 200 nm using a Jasco J-815 CD Spectropolarimeter (Jasco International Co., Ltd., Japan) purged with N2 gas. The purified bacteriocin was diluted 6-fold in 20 mM sodium phosphate buffer (pH 6.0) resulting in the final concentration of ~25 mg/L in the solution. The cell and sample buffer were run as a blank and did not contribute to the spectrum. The spectral measurements were repeated three times at 25 °C using a sample cell with a path length of 1 cm (UV quartz cuvettes, CXA-150-180J, Hellma Analytics, Germany) and the average spectrum was reported. The instrument parameters were as follows; scanning speed 50 nm/min, data pitch 0.1 nm, response time one second and band width 1.00 nm. The spectra were recorded in standard CD mdeg units and were converted to standard unit of molar ellipticity using the theoretical protein concentration. The CD data was used to predict the overall secondary structure of the bacteriocin by using the K2D3 online tool [88].

### 4.10 *De novo* LC-MS/MS peptide sequencing

A *de novo* sequencing approach was used to identify the peptide sequence of the purified bacteriocin peptide using an ESI-LC-MS/MS technique at the Bio21 institute proteomics facility (University of Melbourne, Australia). *L. plantarum* B21 purified bacteriocin was analysed using an LTQ™ Orbitrap Elite ETD (Thermo Scientific, USA) coupled to an UltiMate 3000 RSLCnano System (Dionex, Thermo Scientific, USA). The nanoLC system was equipped with an Acclaim™ Pepmap™ 100 C_18_ nano-trap column (1 × 2 cm, 5 μm particle size, Thermo Scientific, USA) and an Acclaim™ Pepmap™ 100 C_18_ analytical column (length 15 cm, 5 μm particle size, Thermo Scientific, USA). 2 μL of the bacteriocin sample was loaded onto the trap column at 3% (v/v) ACN (Merck, USA) containing 0.1% (v/v) formic acid (Sigma-Aldrich, USA) for 5 min before the enrichment column was switched in-line with the analytical column. An initial run on the LTQ™ Orbitrap Elite ETD mass spectrometer (Thermo Scientific, USA) was set to operate in data-dependent mode, where subsequent MS/MS spectra was acquired for the top five peaks by collision induced dissociation (CID) activation, followed by higher-energy collision dissociation (HCD). To improve the MS/MS information a targeted run was setup where the precursor (with an isolation width of 4 Da) was subject to both CID and HCD at three different energy levels (CID: 26, 32, 36, and HCD: 24, 26, 30) and activation Q of 0.25 (CID) or activation time of 0.1 ms (HCD). The resulting fragment ion spectra were recorded in the Orbitrap at a resolution of 240,000. The spectrum produced from HCD at 26 eV was submitted to MASCOT.

### 4.11 Genetic analysis of bacteriocin gene cluster

Sequence tags were extracted manually from the spectrum resulting from the *de novo* LC-MS/MS peptide sequencing and BLAST against the six-frame translation of the total *L. plantarum* B21 Illumina genome data (GenBank Accession No. CP010528). The DNA sequence of the bacteriocin was found to be located on a native plasmid designated as pB21AG01 (GenBank Accession No. CP025732). Plasmid annotation was performed using Rapid Annotation using Subsystem Technology (RAST) (http://rast.nmpdr.org/) [82] to identify the gene cluster involved in the production of the bacteriocin. All open reading frames (ORFs) identified were then searched against NCBI database using BLAST (http://www.ncbi.nlm.nih.gov) [83] to identify genes associated with bacteriocin production.

### 4.12 Multiple sequence alignment and phylogeny

Initially, signal sequences were removed from experimentally-characterised circular bacteriocins. All members of family ii were permuted by 3 residues (moving the final 3 residues to the beginning of the sequence) to improve the alignment between the two families. As these sequences are circular there is no N or *C terminus*, therefore permutation allowed an improved alignment. Plantacyclin B21AG was aligned against other experimentally-confirmed circular bacteriocins using Clustal Omega [89] (https://www.ebi.ac.uk/Tools/msa/clustalo/) and exported to fasta format andused as input for RAxML (raxmlHPC-PTHREADS-SSE3 version 8.2.10) [90] using the following parameters: -T 4 -f a -x 285 -m PROTGAMMABLOSUM62 -p 639 –N 1000 –O The bipartitions output file was used in FigTree version 1.4.4 (http://tree.bio.ed.ac.uk/software/figtree/) for viewing/manipulation.

## Declarations

### Author contribution statement

Aida Golneshin: Conceived and designed the experiments; Performed the experiments; Analyzed and interpreted the data; Wrote the paper.

Mian-Chee Gor: Performed the experiments; Analyzed and interpreted the data; Wrote the paper.

Ben Vezina: Analyzed and interpreted the data; Wrote the paper.

Nicholas Williamson: Conceived and designed the experiments; Performed the experiments; Analyzed and interpreted the data.

Thi Thu Hao Van: Performed the experiments; Analyzed and interpreted the data.

Bee K. May: Contributed reagents, materials, analysis tools or data.

Andrew T. Smith: Conceived and designed the experiments; Wrote the paper.

### Funding statement

This work was supported by 10.13039/501100001780RMIT University, Melbourne, Australia.

### Competing interest statement

The authors declare no conflict of interest.

### Additional information

No additional information is available for this paper.

## Figures and Tables

**Sup Figure 5:**
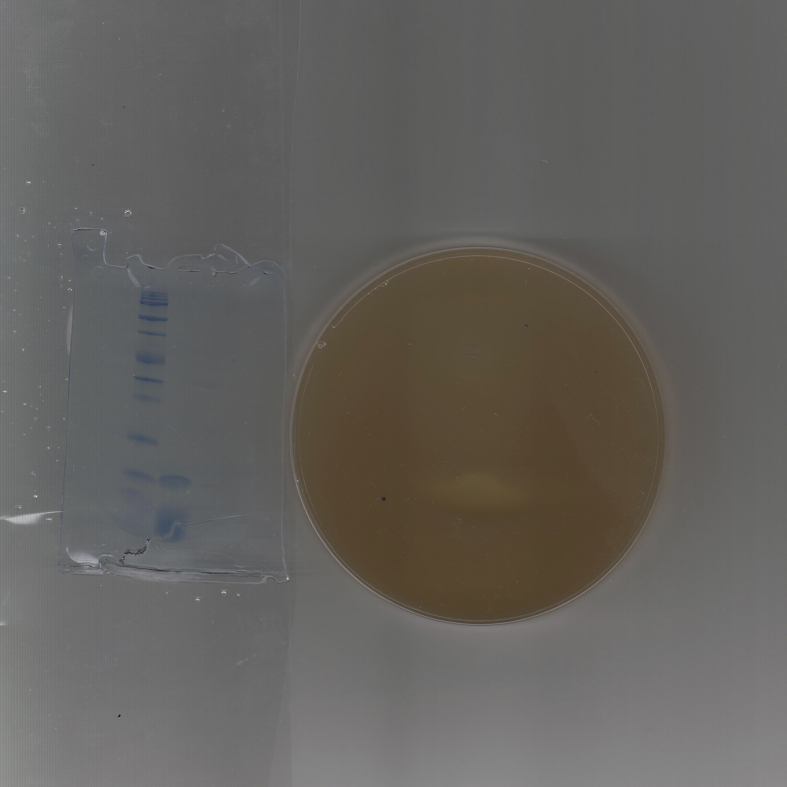
Original gel picture found in Figure 3.

## Appendix A Supplementary data

Sup Data 1.fasta Sup Data 1.fasta

Sup Table 1 Sup Table 1
