# Supplementary material for: Discovery and characterisation of circular bacteriocin plantacyclin B21AG from Lactiplantibacillus plantarum B21
Source: Heliyon. 2020 Aug 21;6(8):e04715. doi: 10.1016/j.heliyon.2020.e04715 (PMC7452424; doi:10.1016/j.heliyon.2020.e04715)
Supplement: Sup Table 1 [file mmc2.docx]

| Purification steps | Volume (mL) ^a^ | Activity (AU/mL) | Total activity (AU) ^b^ | Protein (mg/mL) ^c^ | Total protein (mg) ^d^ | Specific activity (AU/mg protein) ^e^ | Yield (%) f | Purification fold ^g^ |
| --- | --- | --- | --- | --- | --- | --- | --- | --- |
| CFS | 165 | 800 | 132,000 | 10.82 | 1,785.63 | 74 | 100 | 1 |
| Butanol extraction (concentrated CFS) | 7.2 | 12,800 | 92,160 | 6.53 | 47.02 | 1,960 | 70 | 27 |
| Gel filtration (NAP10 desalting) | 1.5 | 25,600 | 38,400 | 1.91 | 2.86 | 13,438 | 29 | 182 |
| FPLC (Concentrated purified bacteriocin) | 0.2 | 128,000 | 25,600 | 0.20 | 0.04 | 630,542 | 19 | 8521 |

Table S1. Purification of *L. plantarum* B21 bacteriocin protein

^a^ Only a part of the concentrated CFS was used in the purification process. The purification table volumes are presented accordingly.

^b^Total activity was determined by multiplying the volume and the activity. ^c^ Protein concentration was determined by BCA assay. ^d^ Total protein was determined by multiplying volume and the protein. ^e^ Specific activity is the activity units divided by protein concentration. ^f^ The yield is the total activity as the percentage of the initial total activity. ^g^ Purification fold was calculated based on specific activity.
